# Environmental DNA for freshwater fish monitoring: insights for conservation within a protected area

**DOI:** 10.7717/peerj.4486

**Published:** 2018-03-06

**Authors:** Sara Fernandez, Miguel M. Sandin, Paul G. Beaulieu, Laura Clusa, Jose L. Martinez, Alba Ardura, Eva García-Vázquez

**Affiliations:** 1 Department of Functional Biology, University of Oviedo, Oviedo, Asturias, Spain; 2 Sorbonne Université, Station Biologique de Roscoff, UMR7144, Roscoff, France; 3 Tighe & Bond, Trout Unlimited, USA; 4 Scientific-technical services, University of Oviedo, Oviedo, Asturias, Spain

**Keywords:** PCR-RFLP, River, *Oncorhynchus mykiss*, Genetics, qPCR, Protected areas, Environmental DNA, *Salmo trutta*

## Abstract

**Background:**

Many fish species have been introduced in wild ecosystems around the world to provide food or leisure, deliberately or from farm escapes. Some of those introductions have had large ecological effects. The north American native rainbow trout (*Oncorhynchus mykiss* Walbaum, 1792) is one of the most widely farmed fish species in the world. It was first introduced in Spain in the late 19th century for sport fishing (Elvira 1995) and nowadays is used there for both fishing and aquaculture. On the other hand, the European native brown trout (*Salmo trutta* L.) is catalogued as vulnerable in Spain. Detecting native and invasive fish populations in ecosystem monitoring is crucial, but it may be difficult from conventional sampling methods such as electrofishing. These techniques encompass some mortality, thus are not adequate for some ecosystems as the case of protected areas. Environmental DNA (eDNA) analysis is a sensitive and non-invasive method that can be especially useful for rare and low-density species detection and inventory in water bodies.

**Methods:**

In this study we employed two eDNA based methods (qPCR and nested PCR-RFLP) to detect salmonid species from mountain streams within a protected area, The Biosphere Reserve and Natural Park of Redes (Upper Nalón Basin, Asturias, Northern Spain), where brown trout is the only native salmonid. We also measured some habitat variables to see how appropriate for salmonids the area is. The sampling area is located upstream impassable dams and contains one rainbow trout fish farm.

**Results:**

Employing qPCR methodology, brown trout eDNA was detected in all the nine sampling sites surveyed, while nested PCR-RFLP method failed to detect it in two sampling points. Rainbow trout eDNA was detected with both techniques at all sites in the Nalón River’ (n1, n2 and n3). Salmonid habitat units and water quality were high from the area studied.

**Discussion:**

In this study, a high quantity of rainbow trout eDNA was found upstream and downstream of a fish farm located inside a Biosphere Reserve. Unreported escapes from the fish farm are a likely explanation of these results. Since salmonid habitat is abundant and the water quality high, the establishment of rainbow trout populations would be favored should escapes occur. Environmental DNA has here proved to be a valuable tool for species detection in freshwater environments, and the probe-based qPCR highly sensitive technique for detection of scarce species. We would recommend this method for routine monitoring and early detection of introduced species within natural reserves.

## Introduction

Introduced fish species affect recipient ecosystems inducing changes in behaviour, distribution and abundance of native species, as well as affecting ecosystem functioning following the decrease of their favoured prey species (Strayer, 2010). An important source of fish introductions is inadvertent escapes from fish farms and aquaculture facilities (Naylor et al., 2005; Consuegra et al., 2011). Salmonids are native to the Northern Hemisphere but have been introduced and farmed worldwide causing disturbances to native species, especially in the Southern Hemisphere (Townsend, 2003; Valiente et al., 2007; Consuegra et al., 2011). Introduced salmonids interact with local fish in many ways: inducing behaviour changes (Cambray, 2003; Fausch, 2007; Landergren, 1999; Townsend, 2003; Wissinger et al., 2009); competing for food resources (Mooney & Cleland, 2001; Eby et al., 2006; Buria et al., 2010); causing changes in trophic webs (e.g., Diehl et al., 2000; Mclntosh et al., 1996), and others.

Rainbow trout (*Oncorhynchus mykiss* Walbaum, 1792), a North American salmonid, is one of the most widely introduced fish species in the world and the most important freshwater fish exploited in aquaculture (Stanković et al., 2015). It is a well-known top predator in freshwater ecosystems (Oscoz et al., 2005; Fausch, 2007; Stanković et al., 2015). Rainbow and brown trout use similar resources and can thus compete for food or space (Oscoz et al., 2005). Introduced rainbow trout negatively impacts on European native brown trout (*Salmo trutta* L. 1758) populations, especially on those inhabiting small streams (Landergren, 1999). Rainbow trout is present in many European streams (Stanković et al., 2015). It was first introduced in Spanish waters in the late 19th century for sport fishing, and now is farmed there as well (Elvira, 1995). A few years ago, there was no evidence of self-sustaining rainbow trout populations in Spain (Doadrio, 2001), but it is expected that they will occupy river areas close to fish farms if escapes occur (Carss, 1990).

The native brown trout, although described as invasive in areas of the Southern Hemisphere, is catalogued as vulnerable in Spain because populations had been reduced by 20% at the end of the 20th century (Doadrio, 2001). The causes of its decrease are a combination of habitat losses, genetic introgression from introduced central European brown trout lineages, exotic species introductions and overfishing (Doadrio, 2001).

For the reasons above, the evaluation of native and invasive fish populations is essential in monitoring the health of an ecosystem (Arlinghaus, 2006). This can be difficult, especially when their density is low, using conventional sampling methods such as electrofishing and netting (Clusa et al., 2017). Moreover, these types of sampling encompass some mortality (Snyder, 2004) and are not suitable for some ecosystems such as those located within protected areas. On the other hand, environmental DNA (eDNA), defined as the genetic material obtained directly from environmental samples such as soil, sediment, water, etc. (Thomsen & Willerslev, 2015), can enable the detection of species that can be elusive or difficult to sample. This technique is commonly used today for species detection (Dejean et al., 2012; Ardura et al., 2015; Ardura et al., 2016; Clusa et al., 2016; Devloo-Delva et al., 2016) and biodiversity inventory (Zaiko et al., 2015; Civade et al., 2016). It is a non-invasive sampling technique that avoids distress to the fish allowing for compliance with the European Code of Conduct for Research Integrity (Drenth, 2011).

Amongst the methods of molecular analysis of eDNA, quantitative PCR (qPCR) has been shown to be highly sensitive, particularly when determining the presence of rare or low-density species (Laramie, Pilliod & Goldberg, 2015). An alternative method is nested PCR, sometimes coupled with RFLP (Restriction Fragment Length Polymorphism), for example that described in Clusa et al. (2017) as a sensitive tool for detecting several salmonid species in water samples. In this study, the objectives were two-fold. On the technical side we have compared the sensitivity of the two methodologies (qPCR and nested PCR + RFLP) to detect eDNA of salmonids from running waters. On the ecological side we have employed these methods to assess possible escapes of farmed rainbow trout in a mountainous protected area, the Biosphere Reserve and Natural Park of Redes (Upper Nalón Basin, Asturias, Northern Spain), where the only native salmonid present is brown trout.

## Methods

### Ethics statement

This project and the sampling carried out in protected spaces was authorized by the entity legally entitled to do so in Spain: the Government of the Asturias Principality, with the permit reference 101/16. The authors adhered to the European Code of Conduct for Research Integrity (Drenth, 2011).

### Study area-upper Nalón Basin

**Figure 1:**
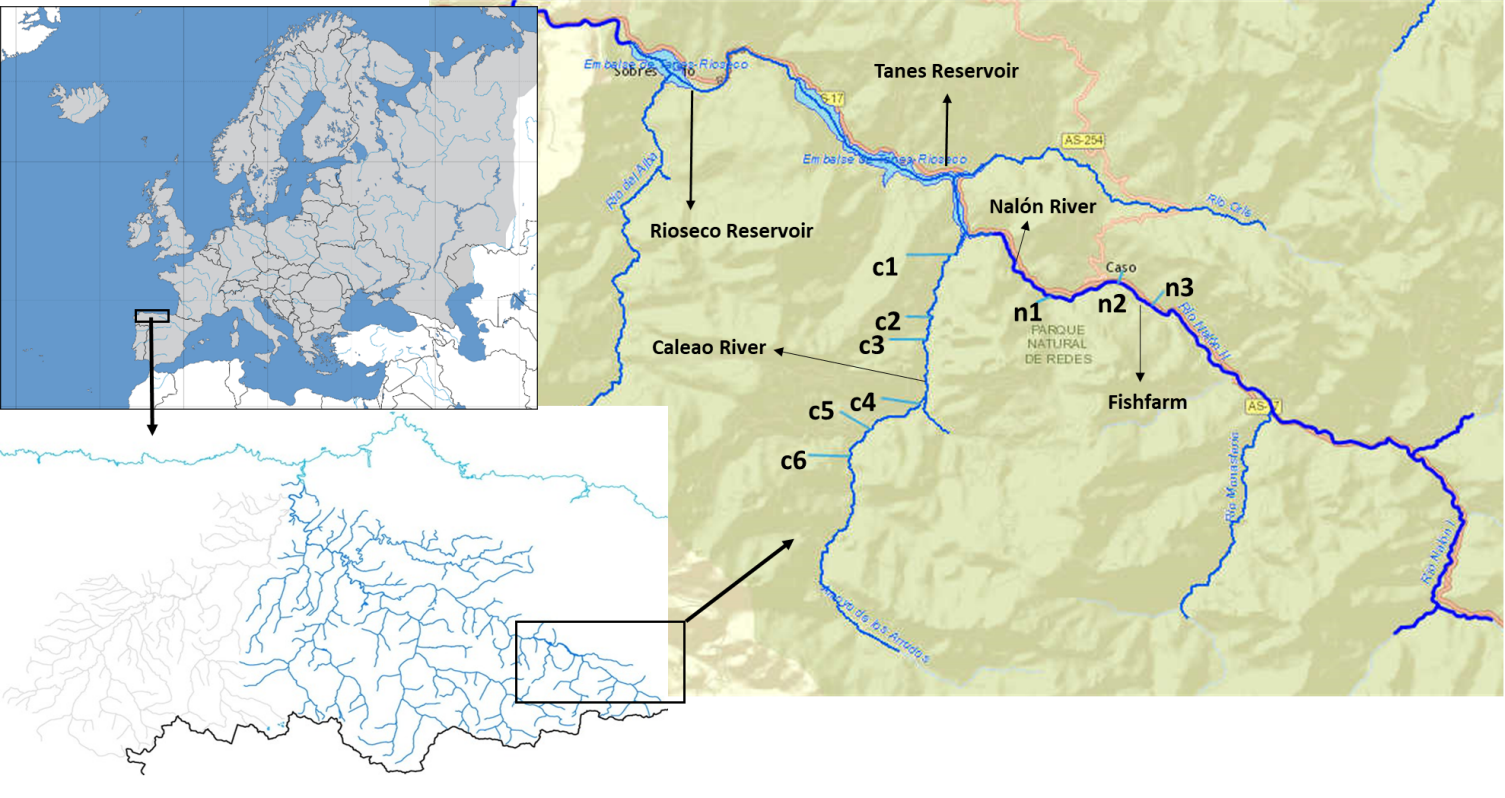
Upper Nalón River Basin. Upper Nalón Basin Map showing sampling points in Nalón River (n1–n3) and its tributary Caleao River (c1–c6) where the study was developed. The impounded areas are marked (Tanes and Rioseco reservoirs).

The Upper Nalón Basin is located in the central part of the region of Asturias (Bay of Biscay, Spain). As part of the UNESCO (United Nations Educational, Scientific and Cultural Organization) Biosphere Reserve and Natural Park of Redes, it has a high faunal diversity (García-Ramos et al., 2006). In the streams (river headwaters) brown trout (*Salmo trutta)* are the only native fish species, because two consecutive impassable dams (Fig. 1) impede the arrival of native migratory European eel (*Anguilla anguilla*) and Atlantic salmon (*Salmo salar*), which occur downstream (Juanes et al., 2011). There is a rainbow trout fish farm located in one of the headwater streams in a zone denominated Veneros (Fig. 1), but escapes have not been reported and rainbow trout individuals were not recorded within the studied streams so far.

### Field methods and macroinvertebrates sampling

Sampling took place in November 2015. Targeted sampling areas were typical juvenile habitat: shallow, well oxygenated and with moderate current velocity. At the sampling time of the year, in the particular river zones sampled trout juveniles around one-year-old are expected to be by far the most abundant life stage (Alonso, Gortázar & García de Jalón, 2010). Two sampling points were chosen along the upstream Nalón River (points n1, n2) and six on one of its head tributaries, Caleao river (c1 to c6; Fig. 1). The two streams are connected at the tail end of Tanes reservoir (Fig. 1). In each sampling point, the habitat was characterized based on the official protocol of the Spanish Ministry of Environment, Feeding and Agriculture (Alba et al., 2005), to check if it was appropriate for salmonids. A 20-meter transect was analysed per site. A profile of the substrate was drawn, characterized as percentage of blocks, boulders, gravel, sand and silt. The dominant vegetation type covering the river (river canopy) was identified as well as the shade percentage and continuity. Three measures of depth and width were taken within the 20-meter transect at each sampled site. From these data, the total amount of juvenile salmonid (‘rearing’) habitat units were estimated for each site based on the model described by Juanes et al. (2011). All profile variables were considered to assign a percentage of juvenile’s habitat to each sampling point. Then, it was standardized to 100 m^2^ (estimated rearing units or ERU) ( Table S1).

Physical-chemical water properties were recorded with a Horiba U-50 multimeter at three different points in each sampled site. To minimize the potential for biased data (i.e.: after a storm) abiotic parameters were replicated three times with a seven-day interval between each reading and the average of these three measurements was presented. Measured parameters included water temperature (°C), pH and TDS (Total Dissolved Solids) (Table 1).

**Table 1:** Sampling Stations (November 2015). Physical-chemical features (TEMP: Temperature. TDS: Total Dissolved Solids). ERU: Estimated Rearing Units for Brown Trout. EQR: Ecological Quality Ratio, based on macroinvertebrate families. The ecological state based on macroinvertebrates was classified following the official protocol of Environment, Feeding and Agriculture Ministry (NIPO: 770- 11-308-X), and is highlighted in yellow for Moderate and green for Good (Alba et al., 2005). Detection (Nested PCR-RFLP) and quantification (qPCR) of Trout eDNA.

Station	Local name	Water course	Coordinates	TEMP (°C)	pH	TDS (g/L)	ERU	EQR	Ecological state	Number of trout DNA molecules (qPCR)		Nested PCR-RFLP	
										Rainbow trout DNA molecules	Brown trout DNA molecules	Rainbow trout eDNA presence/ no presence	Brown trout eDNA presence/ no presence
c1	Arrudos	Caleao river	43°08′46.8N 5°24′47.9W	9.07	7.90	0.11	20.09	0.62	Good	0	1,964.85	0	X
c2	Caliao	Caleao river	43°09′11.2N 5°24′24.0W	9.41	8.13	0.11	69.78	0.48	Moderate	0	3,119.54	0	X
c3	Encruceyada	Caleao river	43°09′24.9N 5°23′39.4W	9.60	8.27	0.12	17	0.48	Moderate	0	1,125.65	0	X
c4	Puentepiedra	Caleao river	43°10′12.2N 5°23′32.3W	10.48	8.36	0.13	20.11	0.48	Moderate	0	4,394.81	0	X
c5	NA	Caleao river	43°10′26.8N 5°23′29.2W	10.73	8.44	0.13	27.39	0.48	Moderate	0	2,231.15	0	X
c6	NA	Caleao river	43°11′16.2N 5°23′01.5W	10.92	8.39	0.13	53	0.48	Moderate	0	1,459.83	0	X
n3	Veneros	Nalón river	43°10′35.2N 5°19′55.6W	–	–	–	–	–	–	6,121.93	1,775.02	X	X
n2	El Campu	Nalón river	43°10′51.3N 5°20′27.7W	10.89	8.35	0.12	86.17	0.66	Good	5,456.07	1,679.35	X	0
n1	Cueva Deboyu	Nalón river	43°10′41.0N 5°21′36.3W	10.96	8.38	0.12	75	0.55	Good	94,342.76	2,307.53	X	0

In addition to abiotic measures, macroinvertebrates communities were studied to calculate water quality indices. Individuals were sampled following the kick-net sampling methodology described in the protocol employed (Alba et al., 2005). Collected specimens were identified down to Family level using an identification key (Oscoz, Galicia & Miranda, 2011), and family presence-absence was recorded. Ecological quality ratio (EQR) and the ecological state of each point were calculated based on macroinvertebrates family’s composition, giving a punctuation to each family based on their tolerance to contamination (Alba et al., 2005) (Table 1).

One extra point was selected in Nalón River to collect only water samples (n3). Taken upstream n2 (where a rainbow trout fish farm is located, see Fig. 1), this water sample (n3) was collected 20 m upstream fish farm discharges as a control to discard the possibility that rainbow trout DNA comes only from fish farm runoff.

### Water sample collection, filtration and eDNA extraction

Water samples of 1.5 L each were collected in sterile plastic bottles from the sampling points. Water samples were vacuum-filtered using a Supor^®^-200 Membrane Filter (Pall Corporation, Life Sciences, Ann Arbor, MI, USA) with 0.2 µm pore size. The filtration room was free of external sources of contamination as it was separated from the molecular laboratory. The filtration system was cleaned up with 10% commercial chlorine based-bleach between samples to avoid contamination between sampling points. 1.5 L of Milli-Q water that was previously transported with the rest of the water samples from the field, was filtered at last following the same steps, and was considered as an extra sample to monitor possible field or filtering contaminations. Finally, filters were placed into 15mL tubes using sterile forceps and stored at −20 °C until DNA extraction.

DNA was extracted from filters with PowerWater^®^ DNA Isolation Kit (MoBio Laboratories Inc., Carlsbad, CA) and preserved at −20 °C until further processing. The DNA extractions were conducted under sterile conditions inside a laminar flow PCR-cabinet, following the manufacturer’s instructions. A negative control was added at this step to monitor contaminations during the extraction process.

### Quantitative PCR procedures

Quantitative PCR from eDNA using specific primers has been validated for brown (Gustavson et al., 2015) and rainbow trout (Wilcox et al., 2015) (Table 2). Details about qPCR protocols are included in this section, as recommended by Bustin et al. (2009):

Two specific TaqMan assays were selected as molecular markers: for brown trout, a 61 base pairs (bp) fragment of the mitochondrial cytochrome oxidase I gene (COI: Cytochrome Oxidase Subunit I) (Gustavson et al., 2015); and a 102 bp fragment of the NADH gene for rainbow trout (Wilcox et al., 2015). In silico analysis were done using the Primer Blast application included in the NCBI webpage (Ye et al., 2012) to check the specificity of the markers. No coincidences were found with related nor cohabiting species.

**Table 2:** qPCR molecular markers. TaqMan assays employed in the qPCR analysis for each targeted species. Primers’ and hydrolysis probes’ sequences.

qPCR					
Species	Source	Gene	Primer	Sequence (5′-3′)	Amplicon(bp)
Brown trout	Gustavson et al. (2015)	COI	Forward	TTTTG TTTGGGCCGTGTTAGT	61
			Reverse	TGCTAAAACAGGGAGGGAGAGT	
			Probe	ACCGCCGTCCTCT	
Rainbow trout	Wilcox et al. (2015)	NADH	Forward	AGTCTCTCCCTGTATATCGTC	102
			Reverse	GATTTAGTTCATGAAGTTGCGTGAGTA	
			Probe	6FAM-CCAACAACTCTTTAACCATC-MGBNFQ	

Pre-PCR analyses of eDNA samples were carried out in a room separated from the molecular laboratory where there is no DNA nor tissue samples, inside a flow PCR-cabinet. Negative controls from the field, filtration and extraction processes were included in PCR runs as well as a PCR negative control.

The qPCR (quantitative Polymerase Chain Reaction) runs were performed using 7,900 HT Fast Real-Time PCR System (Life Technologies, Inc., Applied Biosystems, Carlsbad, CA, USA). Amplification reaction mixtures for brown trout included: 10 µl of TaqMan Environmental Master Mix 2.0 (Life Technologies, Carlsbad, CA, USA), 0.4 µl of each Primers (final concentration of 0.2 µM), and 0.4 µl hydrolysis probe (final concentration of 0.2 µM), and DNA template (6 µl of eDNA extracted from water samples, or from 43 ng of tissue DNA), up to a final 20 µl volume. Amplification reaction mixtures for rainbow trout included: 10 µl of TaqMan Environmental Master Mix 2.0 (Life Technologies, Carlsbad, CA, USA), 0.6 µl of Forward primer (final concentration of 0.3 µM), 1.2 µl of Reverse Primer (final concentration of 0.6 µM) and 0.5 µl of hydrolysis probe (final concentration of 0.25 µM) and DNA template (6 µl and 43 ng of eDNA and tissue DNA respectively), also up to a final 20 µl reaction volume.

As positive controls, DNA from tissue samples of each species were extracted with E.Z.N.A.^®^ Tissue DNA Kit (Omega, Bio-Tek, Norcross, GA, USA) following the manufacturer’s instructions. The two molecular markers were tested first on control DNAs. A mixture of control DNAs from rainbow and brown trout at known concentrations were PCR amplified with the two specific markers to check for possible co-amplification or interference between them (Fig. S1). On each qPCR run, a positive control from tissue extractions of the targeted species was also added to monitor PCR inhibition.

PCR amplicons were generated with the two primer sets from tissue DNA in a total volume of 20 µl, including Green GoTaq^®^ Buffer 1X, MgCl_2_, 0.25 mM dNTPS, 0.25 µM of each primer, 4 µl of template DNA and 0.65U of DNA Taq polymerase (Madison, WI, USA). PCR conditions were 95 °C for 5 min, followed by 35 cycles at 94 °C for 30 s, 57 °C for 30 s and 72 °C for 30 s, and a final step of elongation at 72 °C for 10 min.

The PCR amplicons obtained were quantified by fluorimetry using Qubit^®^ dsDNA BR Assay Kit (Thermofisher Scientific, Carlsbad, CA, USA). The amount of DNA was transformed into molecules per µl, calculated from the known base composition of the amplicon sequence. A standard curve was constructed including a serial dilution (from 2.34 × 10^9^ to 2.34 ×10^3^ molecules/µL for rainbow trout and from 6.3 ×10^9^ to 6.3 × 10^3^ molecules/µL for brown trout) and used as reference for DNA molecules quantification in water samples. A dilution series from tissue DNA of each species was done to determine the lowest copy number of target DNA per litre of water detectable from each assay.

All the analyses of tissue and amplicon samples were conducted separately from environmental samples, keeping them away from any source of contamination.

To confirm the correct target species detection, amplicons from some environmental samples were sequenced. Sequencing was carried out in the Sequencing Unit of University of Oviedo’s Scientific-Technical services.

**Table 3:** PCR-RFLP Markers. Primers’ sequences and amplicon’s length employed in the nested PCR assay for rainbow and brown trout eDNA detection.

Nested PCR-RFLP					
	Gene	Primer	Source	Sequence (5′–3′)	Amplicon (bp)
First-PCR	16S	Forward	Clusa et al. (2017)	GCCTGCCCTGTGACTATGG	567
		Reverse	Palumbi et al. (2002)	CCGGTCTGAACTCAGATCACGT	
Nested-PCR		Forward	Zaiko et al. (2015)	AAGACCTGTATGAATGGCATC	377
		Reverse	Zaiko et al. (2015)	TCGATAGGGACTCTGGGAGA	

### Species-specific PCR-RFLP

The nested PCR-RFLP method was developed as described in Clusa et al. (2017) for detecting the two targeted species (brown and rainbow trout). Briefly: a first PCR was carried out to amplify a 567 bp fragment of the 16S rRNA gene, using as forward the 16S-new-F primer designed in the cited research (Table 3), and the reverse 16S-Br universal primer from Palumbi et al. (2002). A nested PCR amplification was then performed with the pair of Salmonidae-specific primers described in Zaiko et al. (2015) (Table 3). The nested PCR product was digested with TaaI and Tru1I FastDigest enzymes (Thermo Fisher Scientific Inc., Waltham, MA, USA) that produce diagnostic band patterns in agarose gel allowing to identify the two targeted species. With TaaI enzyme, brown trout DNA gives two bands of 205 and 272 bp, and with Tru1I, rainbow trout DNA gives a band of 66 bp.

## Results

### Habitat quality measures

The physical-chemical characteristics of the sampling points were within the optimum range for Salmonid juveniles (Raleigh et al., 1984; Raleigh, Zuckerman & Nelson, 1986). The numbers of juvenile salmonid habitat units (ERU) ranged between 17 in c4 and 86.17 in n2 (Table 1).

The ecological state measured from macroinvertebrate communities (EQR) was good at the points from the upper zone of Caleao River and in Nalon River (c6, n1 and n2), and moderate in the rest of the points. Detailed results of macroinvertebrate families along sampling stations are summarized in Table S2.

### qPCR assays

Assays of control DNAs provided the same quantification cycle values in the samples with single species DNA as well as in the mixture DNA samples (17 cycles for rainbow trout marker and 15 cycles for brown trout marker; see Figs. S1A and S1B respectively). Thus, there were no interferences caused by the presence of DNA from the other species (Fig. S1).

Standard curves for brown and rainbow trout fitted the equations *y* =  − 3.819*x* + 45.141, *R*^2^ = 0.999 and *y* =  − 3.3128*x* + 39.2113, *R*^2^ = 0.999 respectively. The lowest detectable number of copies was 7 ×10^2^ and 2.6 ×10^3^ molecules per liter for brown and rainbow trout assays respectively. From eDNA, negative controls were clean as expected i.e., no evidence of amplification. As positive amplification was obtained from all eDNA extraction samples for brown trout assay (see below), PCR inhibition testing was not performed.

Values of quantification obtained from river water samples varied from 94.34 × 10^2^ to 11.26 × 10^2^ trout DNA molecules (Table 1). Brown trout eDNA was found from all nine sampling sites. Rainbow trout eDNA occurred from the three points located in Nalón River (Table 1), one 20 m upstream (Veneros) and two downstream from the rainbow trout fish farm located in that river. The amount of rainbow trout eDNA molecules was considerably higher than that of brown trout in the Nalón River sampling points (n1 to n3), especially in n1 where the highest amount (94342.76 eDNA molecules) was found. This point is located in the lowest zone of the sampling area (Fig. 1).

### PCR-RFLP

**Figure 2:**
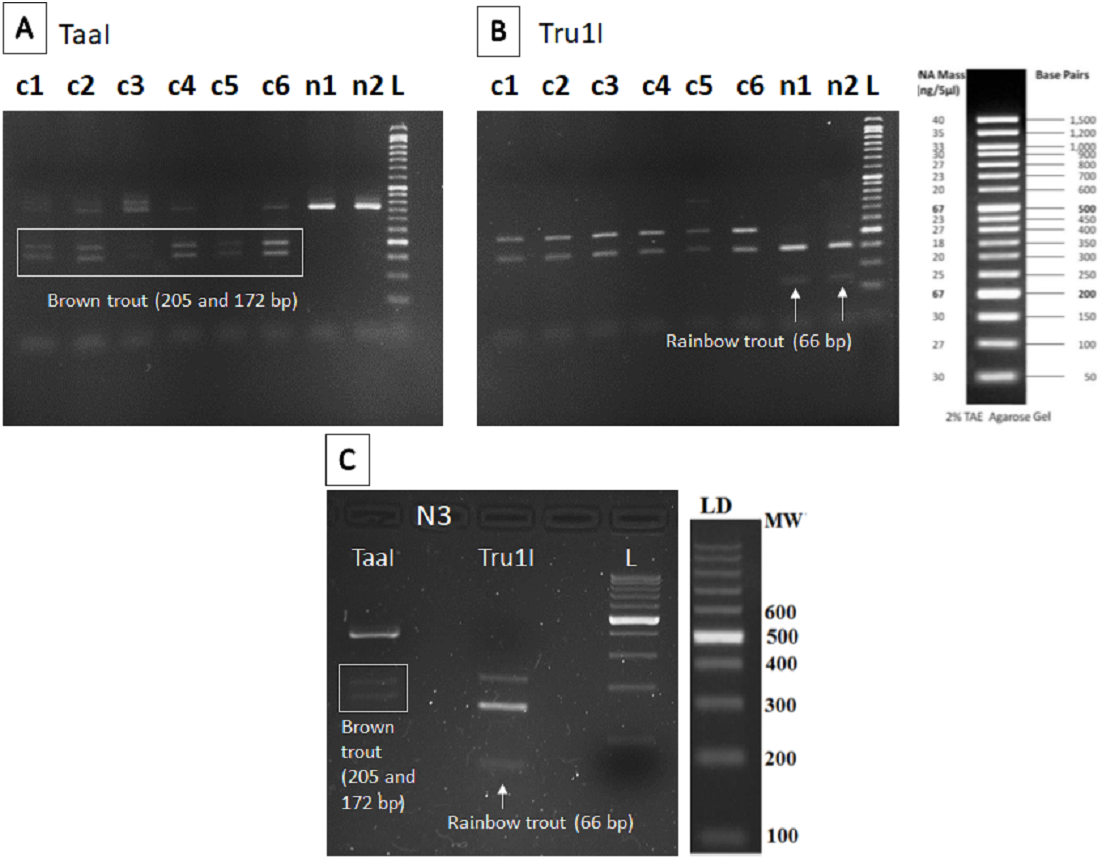
RFLP results of brown and rainbow trout. Diagnostic fragments of each species are indicated (c1–c6: sampling points in Caleao River; n1–n3: sampling points in Nalón River; L, Ladder to estimate fragment size; TaaI and Tru1I: restriction enzymes). (A) Restriction pattern obtained for brown trout detection (205 and 172 bp) with TaaI enzyme digestion from eDNA samples. (B) Restriction pattern obtained for rainbow trout (66 bp) with TruI enzyme digestion from eDNA samples. (C) Restriction patterns obtained with TaaI and Tru1I enzymes characteristic of brown (205 and 172 bp) and rainbow (66 bp) trout respectively, found in sampling Point n3, Veneros, from eDNA sample (upstream fish farm discharges).

The restriction patterns within the fragment amplified provided specific bands for brown trout (205 and 172 bp) after digestion with TaaI enzyme in all sampling points except n1 and n2 (Fig. 2A), where only the specific band for rainbow trout (66 bp) was found after digestion with Tru1I enzyme (Fig. 2B). Rainbow trout typical RFLP pattern was also found in n3 after Tru1I digestion (Fig. 2C).

## Discussion

In this study, we have detected eDNA signals of rainbow trout on running waters that can be interpreted as fish farm escapes within a protected area. As in other studies, environmental DNA again proved its value as a tool for species detection in freshwater environments (Jerde et al., 2011). Moreover, the probe-based qPCR was confirmed to be a highly sensitive technique that has the potential to offer quantification and biomass estimations (Gustavson et al., 2015). Although the PCR-RFLP technique employed here had demonstrated its sensitivity for salmonids detection in water samples (Clusa et al., 2017), it was not able to detect brown trout DNA in all the sampling points of the current study (n1 and n2), where it was detected from qPCR. The high amount of rainbow trout eDNA in these two sampling points, clearly more abundant than brown trout DNA, was a likely cause of the false brown trout negative found using PCR-RFLP technique. That technique is based on initial PCR amplification of a salmonids-specific DNA fragment which is the template for enzymatic digestion; the initial PCR amplification of the much more abundant rainbow trout DNA probably led to very weak undetectable brown trout bands (at least on agarose gels). PCR-RFLP technique has also another limitation compared with qPCR, which is the probability of cross-contamination, more probable when using a gel-based system rather than an enclosed system as qPCR is.

Indeed, the results obtained using PCR-RFLP reinforced the conclusion of widespread rainbow trout occurrence in the upper Nalón River (n1, n2 and n3) found from qPCR. The two methods have detected rainbow trout DNA from running waters upstream of an impassable dam, where rainbow trout is not expected to occur naturally. Moreover, fish farm escapes have not been officially reported in this area, nor have established populations been documented. There are rainbow trout fish farms downstream of Tanes reservoir, but it is an impassable barrier, thus arrival of escapes from downstream fish farms is impossible. Unreported fish farm escapes within the Biosphere Reserve, or DNA runoff from the fish farm (not individuals) would be alternative explanations. It is also possible that eDNA comes from predator transfers via depositing of carcasses or defecation (Merkes et al., 2014), but much more improbable. The occurrence of rainbow trout individuals (not just trout DNA from fish farm water discharges or runoffs) was strongly suggested here from rainbow trout DNA obtained in the sample taken as a control upstream of the fish farm drainage (n3), because runoff goes with the river current and floating DNA cannot move upstream. Thus, unreported escapes were a likely explanation of these results, which show how important is the location of sampling points in eDNA studies from running waters (Deiner & Altermatt, 2014; Jane et al., 2015). In theory, if escapes occur the escapees may interact with native brown trout inside the protected area, where the introduction of exotic species is strictly prohibited from Spanish legislation (BOPA Decree 48/2006, of 18 of May, implementing the Spanish Law 5/1991, of 5 of April, for Protection of Natural Spaces).

One of the problems of eDNA-based methodology is the possible occurrence of false positives (Beja-Pereira et al., 2009). However, this is likely not applicable to the present study. Negative controls during samples transport and processing were carried out to monitor possible contamination. Possible interferences of species-specific qPCR on DNA mixtures, that may happen in waters containing diverse communities, were also tested *in vitro* and discarded. We verified that there were neither interferences nor co-amplifications between the DNA from the two targeted species, which validates the use of the selected markers for eDNA detection and quantification where the two target species are cohabiting rivers. This is an innovation over previous studies based on eDNA where only one species was targeted (Gustavson et al., 2015; Wilcox et al., 2015). In the nested PCR-RFLP assays, cross-contamination was carefully prevented, as described in Clusa et al. (2017). All our negative sampling and extraction controls were clean; thus contamination can be reasonably ruled out in this study.

The results of the present study could be taken as an alert signal of the presence of rainbow trout in the ecosystem. Although the presence of other exotic species is documented along Nalón River, the information about rainbow trout occurrence in the ecosystem is clearly insufficient. It is generally believed that there are no self-sustaining rainbow trout populations in Spain, but there are no specific studies about its naturalization (Stanković et al., 2015). The methodology employed in this study could serve for a wide and systematic monitoring of this species from all the Iberian rivers basins. One of the benefits of eDNA is allowing to monitor more sites in a faster and cheaper manner (Deiner et al., 2016; Bista et al., 2017), screening environments for potential species from which ground truthing (i.e., electrofishing or netting in the present case) can be conducted later to verify. The river part studied here exhibits a good habitat for salmonids (Juanes et al., 2011). The sampling points where rainbow trout eDNA was found had 161.17 ERUs and the best water quality. Since salmonids habitat is abundant and the water quality high, if escapes of rainbow trout occurred the species establishment would be favoured by those good conditions. This would have both direct and indirect impacts at multiple ecological levels (Mack et al., 2009 Stanković et al., 2015), and would be an enormous threat for the protected ecosystem because the impacts of invasive fish propagate rapidly beyond the habitat initially occupied (Baxter et al., 2004). One of the possible consequences could be the fragmentation of native fish populations (Fausch, 2007). Brown trout populations would be especially affected in this case because River Nalón is already dammed, and upstream populations are forcedly isolated from downstream settings. On the other hand, the introduction of farmed salmonids in the wild (escapes or deliberate releases) is often followed by interspecific hybridization with wild individuals, due to altered behavior of farmed fish (e.g., Horreo et al., 2014; Horreo et al., 2018). Since the survival of brown trout x rainbow trout hybrids is extremely low (Scheerer & Thorgaard, 1983), if hybridization happened the reproductive potential of native brown trout would be diminished. Considering all the risks above together, and the fact that small and isolated brown trout populations are especially vulnerable (Mooney & Cleland, 2001), their conservation within the protected area might be endangered if rainbow trout escapes are not carefully controlled.

### Conclusion and implications for conservation

From our results, and supporting Gustavson et al. (2015), qPCR is more effective for species detection when the eDNA abundance is low or if the number of eDNA molecules of two targeted species are very different. The use of qPCR for species detection in those cases would be recommended.

This is an example of the potential of eDNA for investigating the distribution of native and exotic fish in running waters. In this case, it served for surveying trout species inside a protected area, where sampling using conventional methodology (i.e., electrofishing or netting) should be disregarded as much as possible so as to not disturb vulnerable communities from upstream mountain landscapes. Therefore, we would recommend this strategy for routine monitoring and early detection of exotic species within natural reserves. Although the results support the occurrence of undeclared exotic rainbow trout, they should be confirmed by independent observations and conventional standard monitoring, to sample individuals and to check if rainbow trout presence is sporadic or on the contrary it reproduces in the river. We consider that a stricter control of the fish farm would be recommended, and efficient containment measures of farmed fish should be taken to prevent escapes.

##  Supplemental Information

10.7717/peerj.4486/supp-1 Supplemental Information 1 Supplemental files

10.7717/peerj.4486/supp-2 Figure S1 Amplification Plots of control DNAs **Plot A:** Amplification plot from rainbow trout assay, lines from two samples (one with rainbow trout DNA and the other with the same amoun of rainbow and brown trout DNA) are showed giving the same value for quantification cycles. **Plot B:** Amplification plot from brown trout assay, lines from two samples (one with rainbow trout DNA and the other with with the same amount of rainbow and brown trout DNA) are showed giving the same value for quantification cycles.

## Additional Information and Declarations

### Field Study Permissions

The following information was supplied relating to field study approvals (i.e., approving body and any reference numbers): This project and the sampling carried out in protected spaces were authorized by the entity legally entitled to do so in Spain: The Government of the Asturias Principality, with the permit reference 101/16.

### Data Availability

The following information was supplied regarding data availability: The raw data are provided in the Supplemental Files.
