# Supplementary material for: Environmental DNA for freshwater fish monitoring: insights for conservation within a protected area
Source: PeerJ. 2018 Mar 6;6:e4486. doi: 10.7717/peerj.4486 (PMC5844247; doi:10.7717/peerj.4486)
Supplement: Supplemental Information 1 [file peerj-06-4486-s001.docx]

1. **Supplemental information.**

| Sampling point | Length (m) | Width (m) | Area (m2) | %Juvenile habitat | ERU | ERU/100m^2^ |
| --- | --- | --- | --- | --- | --- | --- |
| C1_1 | 7 | 7.2 | 50.4 | 0.2 | 10.08 | 20.09 |
| C1_2 | 7 | 7 | 49 | 0 | 0 |  |
| C1_3 | 7 | 7.1 | 49.7 | 0.4 | 19.88 |  |
| Total_C1 |  |  | 149.1 |  | 29.96 |  |
| C2_1 | 7 | 6 | 42 | 0.25 | 10.5 | 69.78 |
| C2_2 | 7 | 6.06 | 42.42 | 0.85 | 36.057 |  |
| C2_3 | 7 | 7 | 49 | 0.95 | 46.55 |  |
| Total_C2 |  |  | 133.42 |  | 93.107 |  |
| C3_1 | 7 | 4.25 | 29.75 | 0.5 | 14.875 | 17.00 |
| C3_2 | 7 | 8.5 | 59.5 | 0.1 | 5.95 |  |
| C3_3 | 7 | 4.75 | 33.25 | 0 | 0 |  |
| Total_C3 |  |  | 122.5 |  | 20.825 |  |
| C4_1 | 7 | 6 | 42 | 0.1 | 4.2 | 20.11 |
| C4_2 | 7 | 12 | 84 | 0 | 0 |  |
| C4_3 | 7 | 12.13 | 84.91 | 0.45 | 38.2095 |  |
| Total_C4 |  |  | 210.91 |  | 42.4095 |  |
| C5_1 | 7 | 4.6 | 32.2 | 0.35 | 11.27 | 27.39 |
| C5_2 | 7 | 7.2 | 50.4 | 0.2 | 10.08 |  |
| C5_3 | 7 | 7 | 49 | 0.3 | 14.7 |  |
| Total_C5 |  |  | 131.6 |  | 36.05 |  |
| C6_1 | 7 | 11.5 | 80.5 | 0 | 0 | 53.00 |
| C6_2 | 7 | 12.5 | 87.5 | 0.8 | 70 |  |
| C6_3 | 7 | 16 | 112 | 0.7 | 78.4 |  |
| Total_C6 |  |  | 280 |  | 148.4 |  |
| N1_1 | 7 | 11 | 77 | 0.95 | 73.15 | 86.17 |
| N1_2 | 7 | 9 | 63 | 0.6 | 37.8 |  |
| N1_3 | 7 | 10 | 70 | 1 | 70 |  |
| Total_N1 |  |  | 210 |  | 180.95 |  |
| N2_1 | 7 | 12 | 84 | 0.8 | 67.2 | 75.00 |
| N2_2 | 7 | 10 | 70 | 0.9 | 63 |  |
| N2_3 | 7 | 14 | 98 | 0.6 | 58.8 |  |
| Total_N2 |  |  | 252 |  | 189 |  |

**S1 Table.** Estimated Rearing Units (ERU). %Juvenile habitat calculation based on Juanes *et al.*(2012).

| **Sampling points** | c1 | c2 | c3 | c4 | c5 | c6 | n1 | n2 |
| --- | --- | --- | --- | --- | --- | --- | --- | --- |
|  |  |  |  |  |  |  |  |  |
| **Preferred Preys** |  |  |  |  |  |  |  |  |
| ***O.mykiss* preferences** |  |  |  |  |  |  |  |  |
| Tipulidae | 0 | 0 | 1 | 0 | 0 | 1 | 0 | 0 |
| Stratiomyidae | 0 | 0 | 0 | 0 | 0 | 0 | 0 | 0 |
| Nemouridae | 0 | 0 | 0 | 0 | 0 | 0 | 1 | 0 |
| ***S.trutta* preferences** |  |  |  |  |  |  |  |  |
| Limnoniidae | 0 | 1 | 0 | 0 | 0 | 0 | 1 | 0 |
| Simuliidae | 0 | 1 | 1 | 0 | 0 | 1 | 0 | 0 |
| Perlidae | 1 | 1 | 1 | 0 | 1 | 0 | 0 | 0 |
| Hydropsychidae | 0 | 0 | 1 | 0 | 0 | 1 | 1 | 1 |
| **Both salmonids preferences** |  |  |  |  |  |  |  |  |
| Chironomidae | 1 | 0 | 1 | 1 | 1 | 1 | 1 | 0 |
| Heptagenidae | 1 | 1 | 1 | 1 | 1 | 0 | 1 | 0 |
| Baetidae | 1 | 1 | 1 | 1 | 1 | 1 | 1 | 0 |
| Rhyacophilidae | 0 | 0 | 0 | 0 | 1 | 1 | 0 | 1 |
| **No Preferred Preys** |  |  |  |  |  |  |  |  |
| Planaridae | 1 | 0 | 0 | 0 | 1 | 0 | 0 | 0 |
| Hydracarina | 1 | 1 | 1 | 0 | 1 | 1 | 0 | 0 |
| Hydrophilidae /Hydraenidae | 1 | 1 | 0 | 0 | 1 | 0 | 0 | 0 |
| Gyrinidae | 1 | 0 | 0 | 0 | 0 | 1 | 1 | 0 |
| Ephermerellidae | 1 | 0 | 0 | 0 | 0 | 0 | 1 | 1 |
| Leuctridae | 1 | 0 | 0 | 0 | 0 | 0 | 1 | 0 |
| Perlodidae | 1 | 1 | 1 | 0 | 1 | 0 | 1 | 1 |
| Lepidostomatidae | 1 | 0 | 0 | 1 | 0 | 0 | 1 | 0 |
| Leptoceridae | 1 | 0 | 0 | 1 | 0 | 0 | 0 | 0 |
| Odontoceridae | 1 | 0 | 0 | 1 | 1 | 1 | 1 | 0 |
| Perlidae | 0 | 0 | 0 | 1 | 0 | 0 | 1 | 1 |
| Leuctridae | 0 | 0 | 1 | 1 | 0 | 1 | 0 | 0 |
| Hydropsychidae | 1 | 0 | 0 | 0 | 0 | 0 | 0 | 0 |
| Phyganeidae | 0 | 1 | 0 | 1 | 0 | 0 | 0 | 0 |
| Chloroperlidae | 0 | 1 | 1 | 0 | 1 | 1 | 1 | 0 |
| Ancylidae | 0 | 1 | 0 | 0 | 0 | 0 | 0 | 0 |
| Glossiphonidae | 0 | 1 | 1 | 0 | 0 | 0 | 0 | 0 |
| Lumbricidae | 0 | 1 | 0 | 0 | 0 | 0 | 1 | 0 |
| Dytiscidae | 0 | 1 | 0 | 0 | 0 | 1 | 0 | 1 |
| Limephilidae | 0 | 0 | 1 | 0 | 0 | 0 | 0 | 0 |
| Erpobdellidae | 0 | 0 | 1 | 0 | 0 | 0 | 0 | 0 |
| Elmidae | 0 | 0 | 0 | 1 | 1 | 0 | 0 | 0 |
| Leptophlebidae | 0 | 0 | 0 | 0 | 0 | 1 | 0 | 1 |
| Polycentropoditae | 0 | 0 | 0 | 1 | 1 | 0 | 0 | 0 |
| Sphaeridae | 0 | 0 | 0 | 0 | 0 | 0 | 1 | 0 |
| Tubificidae | 0 | 0 | 0 | 0 | 0 | 0 | 1 | 0 |
| Hydrometridae | 0 | 0 | 0 | 0 | 0 | 0 | 1 | 0 |
| Sericostomatidae | 0 | 0 | 0 | 0 | 0 | 0 | 0 | 1 |
| Goeridae | 0 | 0 | 0 | 0 | 1 | 0 | 0 | 0 |

**S2. Table.** Macroinvertebrates family’s occurrence in sampling stations along River Nalón (1= Presence; 0= No Presence).
