# Supplementary figures and images for: Environmental DNA for freshwater fish monitoring: insights for conservation within a protected area
Source: PeerJ. 2018 Mar 6;6:e4486. doi: 10.7717/peerj.4486 (PMC5844247; doi:10.7717/peerj.4486)

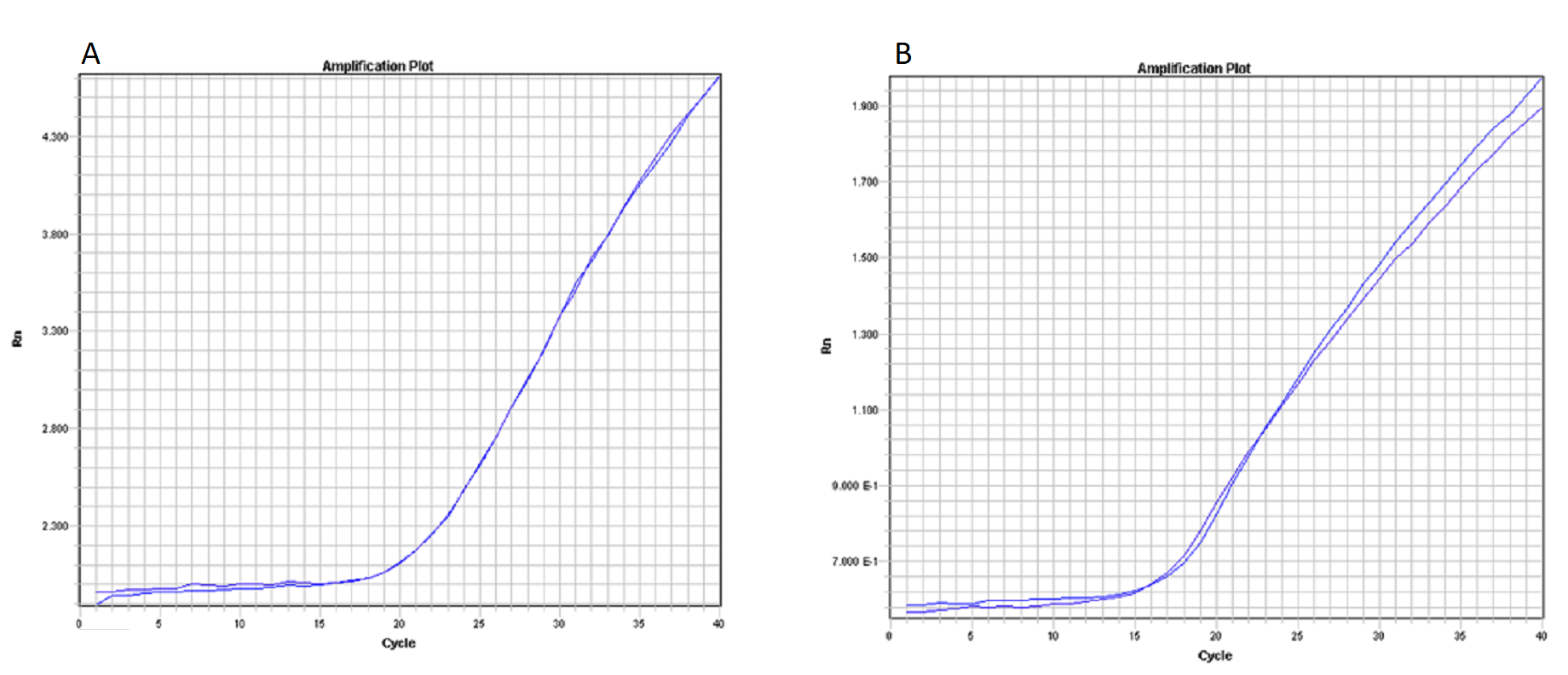

Supplement: Figure S1 — Plot A: Amplification plot from rainbow trout assay, lines from two samples (one with rainbow trout DNA and the other with the same amoun of rainbow and brown trout DNA) are showed giving the same value for quantification cycles. Plot B: Amplification plot from brown trout assay, lines from two samples (one with rainbow trout DNA and the other with with the same amount of rainbow and brown trout DNA) are showed giving the same value for quantification cycles. [file peerj-06-4486-s002.png]
